# The role of epigenetic regulation in pancreatic ductal adenocarcinoma progression and drug response: an integrative genomic and pharmacological prognostic prediction model

**DOI:** 10.3389/fphar.2024.1498031

**Published:** 2024-11-21

**Authors:** Kang Fu, Junzhe Su, Yiming Zhou, Xiaotong Chen, Xiao Hu

**Affiliations:** Department of Hepatobiliary Pancreatic Surgery, The Affiliated Hospital of Qingdao University, Qingdao, China

**Keywords:** pancreatic ductal adenocarcinoma, epigenetic regulation, single-cell RNA sequencing, machine learning, prognostic model, tumor microenvironment, drug sensitivity

## Abstract

**Background:**

Pancreatic ductal adenocarcinoma (PDAC) is a highly lethal malignancy with poor prognosis. Epigenetic dysregulation plays a crucial role in PDAC progression, but its comprehensive landscape and clinical implications remain unclear.

**Methods:**

We integrated single-cell RNA sequencing, bulk RNA sequencing, and clinical data from multiple public databases. Single-cell analysis was performed using Seurat and hdWGCNA packages to reveal cell heterogeneity and epigenetic features. Weighted gene co-expression network analysis (WGCNA) identified key epigenetic modules. A machine learning-based prognostic model was constructed using multiple algorithms, including Lasso and Random Survival Forest. We further analyzed mutations, immune microenvironment, and drug sensitivity associated with the epigenetic risk score.

**Results:**

Single-cell analysis revealed distinct epigenetic patterns across different cell types in PDAC. WGCNA identified key modules associated with histone modifications and DNA methylation. Our machine learning model, based on 17 epigenetic genes, showed robust prognostic value (AUC >0.7 for 1-, 3-, and 5-year survival) and outperformed existing models. High-risk patients exhibited distinct mutation patterns, including higher frequencies of KRAS and TP53 mutations. Low-risk patients showed higher immune and stromal scores, with increased infiltration of CD8^+^ T cells and M2 macrophages. Drug sensitivity analysis revealed differential responses to various therapeutic agents between high- and low-risk groups, with low-risk patients showing higher sensitivity to EGFR and MEK inhibitors.

**Conclusion:**

Our study provides a comprehensive landscape of epigenetic regulation in PDAC at single-cell resolution and establishes a robust epigenetics-based prognostic model. The integration of epigenetic features with mutation profiles, immune microenvironment, and drug sensitivity offers new insights into PDAC heterogeneity and potential therapeutic strategies. These findings pave the way for personalized medicine in PDAC management and highlight the importance of epigenetic regulation in cancer research.

## 1 Introduction

Pancreatic cancer is an extremely dangerous malignant tumor. Despite significant advances in cancer treatment over the past few decades, the prognosis for pancreatic cancer remains poor. Statistics show that the 5-year survival rate for pancreatic cancer patients is only 9% (Rawla et al., 2019). More worryingly, the incidence of pancreatic cancer has been on the rise in recent years. Pancreatic ductal adenocarcinoma (PDAC) is the main type of pancreatic cancer, accounting for over 90% of cases, and is projected to become the second deadliest cancer by 2030 (Nakaoka et al., 2023; Mizrahi et al., 2020). The poor treatment outcomes for PDAC are primarily due to its unique biological characteristics. First, its high metabolic plasticity and adaptability allow it to survive and proliferate rapidly in harsh tumor microenvironments (Bi et al., 2024; Shah et al., 2024). Second, the dense stroma specific to PDAC not only hinders drug penetration but also enables it to evade immune system surveillance (Wilson et al., 2014; Timmer et al., 2021). Third, PDAC exhibits high tumor heterogeneity (Bailey et al., 2016; Wang X. et al., 2023). These characteristics collectively lead to its resistance to traditional treatment methods.

In recent years, two major epigenetic mechanisms - DNA methylation and histone modification - have been recognized as playing crucial roles in the occurrence, progression, and treatment resistance of PDAC. Lomberk et al. elucidated that data from many laboratories have demonstrated that oncogenic mutations in PDAC (such as Kras) lead to downstream signaling events that regulate histone and DNA modifications, partly through direct regulation of histones and histone and DNA modifying enzymes, thereby stimulating cell growth (Lomberk et al., 2019). Cedar et al. and Liu et al. also described the interdependence and crosstalk between DNA methylation and histone modification patterns (Cedar and Bergman, 2009; Liu et al., 2016). DNA methylation primarily occurs on CpG islands and is usually associated with gene silencing (Nishiyama and Nakanishi, 2021). In PDAC, several key tumor suppressor genes, such as CDKN2A (Goodwin et al., 2023), RASSF1A (Amato et al., 2016), and BRCA1 (Lai et al., 2021), have been found to be inactivated due to hypermethylation in their promoter regions, leading to dysregulation of important pathways such as cell cycle regulation, DNA repair, and apoptosis, promoting tumor formation and progression. On the other hand, histone modifications regulate gene expression by altering chromatin structure and transcription factor accessibility. In PDAC, abnormalities in histone acetylation and methylation have been widely reported. For example, overexpression of histone deacetylases (HDACs) leads to silencing of multiple tumor suppressor genes (Schneider et al., 2010), while upregulation of the histone methyltransferase EZH2 is associated with increased invasiveness and metastatic potential of PDAC (Versemann et al., 2022).

To date, although there have been numerous studies on the molecular mechanisms of DNA methylation and histone modification in PDAC, no research has constructed a comprehensive epigenetic regulatory landscape and prognostic model. Clinically, there is still no application of epigenetic regulation in disease stratification and treatment. This study aims to construct an epigenetic regulatory landscape of PDAC through integrated analysis of DNA methylation and histone modification data. By applying advanced machine learning algorithms, single-cell analysis, and other techniques, we will identify key epigenetic regulatory modules and explore their associations with gene expression, signaling pathway activation, and clinical phenotypes. Combining patient clinical follow-up data, we will identify PDAC heterogeneity from an epigenomic perspective and develop a prognostic prediction model based on epigenetic features. This model will not only help identify high-risk patients but may also provide guidance for individualized treatment decisions.

## 2 Materials and methods

**FIGURE 1:**
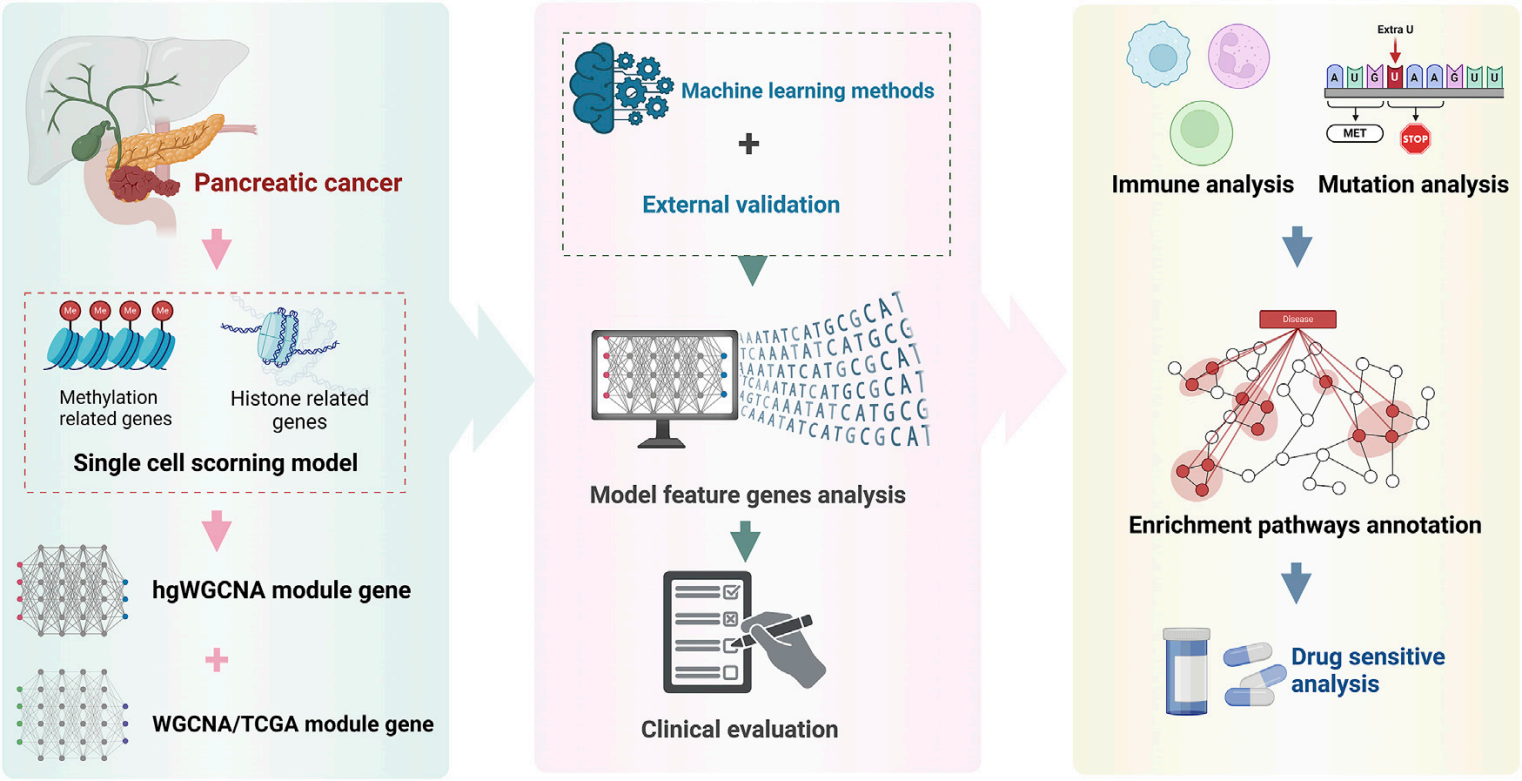
Flow chart.

The flow chart are shown in Figure 1.

### 2.1 Data source

This study integrates multiple public database resources. Specifically, the single-cell sequencing data included was obtained from the Gene Expression Omnibus (GEO) database (https://www.ncbi.nlm.nih.gov/geo/), accession number GSE212966, which contains single-cell sequencing data from 6 PDAC adjacent normal tissue samples and 6 PDAC samples. Additionally, bulk sequencing expression profiles and survival information were sourced from The Cancer Genome Atlas (TCGA) database (https://portal.gdc.cancer.gov/), including gene expression data and corresponding survival information for 179 PDAC samples (Tomczak et al., 2015). The external validation set was obtained from the International Cancer Genome Consortium (ICGC) database (https://dcc.icgc.org/), comprising 234 Canadian samples and 91 Australian samples (Zhang et al., 2019).

Furthermore, in the selection of specific gene sets, we focused on genes related to epigenetics. Histone modification-related genes were primarily sourced from two origins: first, the cancer-related histone modification study reported by Füllgrabe et al. (2011), and second, genes with a histone modification relevance score greater than 20 in the GeneCards database (https://www.genecards.org/). For methylation-related genes, we referenced the research findings of Lee et al. (2020) and supplemented them with genes having a DNA methylation modification relevance score greater than 20 in the GeneCards database.

### 2.2 Single cell analysis and hdWGCNA

The single-cell RNA-seq data in this study was processed using the Seurat package and hdWGCNA package (Stuart et al., 2019; Morabito et al., 2023). First, we read the data in 10X Genomics format from the GSE212966 dataset. Quality control was performed to filter out cells with <100 or >5,000 detected genes, >15% mitochondrial gene content, and <1000 UMI counts. After data filtering and normalization, we performed dimensionality reduction using PCA and UMAP, and integrated different samples using the Harmony algorithm (Cristian et al., 2024). For cell type annotation, we identified specific marker genes for each cell type: cancer cells (EPCAM, KRT19, CEACAM6), fibroblasts (COL1A1, DCN, FAP), endothelial cells (PECAM1, VWF, CDH5), macrophages (CD68, CD163, CSF1R), T cells (CD3D, CD8A, CD4), and B cells (CD79A, CD19, MS4A1). Subsequently, we applied clustering analysis and calculated gene expression differences of the corresponding clusters for cell type annotation. To construct epigenetic scores, we used the ssGSEA method (Chen et al., 2022) to calculate enrichment scores for histone gene sets and methylation gene sets for each cell, and divided cells into high-score and low-score groups based on the median score. On one hand, we performed differential expression analysis using Seurat’s FindAllMarkers function with a logfc threshold of 0 and a minimum percentage of 0.35. On the other hand, we applied the hdWGCNA package to construct weighted gene co-expression networks, determine the optimal soft threshold parameters, identify co-expression modules and hub genes. By calculating module characteristic genes and module connectivity, we analyzed the relationships between modules and cell types and phenotypes, thereby comprehensively revealing the expression patterns of histone modifications and DNA methylation in different cell types and their potential biological functions.

### 2.3 Weighted gene co‐expression network analysis (WGCNA)

The Weighted Gene Co-expression Network Analysis (WGCNA) method was used to further explore the association between epigenetics and PDAC (Langfelder and Horvath, 2008). We calculated histone modification and DNA methylation scores for samples using the ssGSEA algorithm with the Gaussian kernel. Subsequently, we extracted gene expression data for PDAC samples from the TCGA database and selected genes related to histone modification and DNA methylation for WGCNA analysis. By determining the optimal soft threshold parameters (power = 5 for both histone modification and DNA methylation analyses), we constructed gene co-expression networks using the unsigned TOM type with a minimum module size of 50 genes. We identified biologically significant gene modules by setting the merge cut height to 0.15. Notably, we found that histone modification-related genes were mainly enriched in the grey module, while DNA methylation-related genes were primarily enriched in the brown module. We further analyzed the relationships between these modules and sample characteristics (such as histone modification scores and DNA methylation scores) using Pearson correlation. Through correlation analysis of Module Membership and Gene Significance, we identified key genes in each module. The correlation between module membership and gene significance for the grey module (histone modification) and brown module (DNA methylation) was visualized using scatter plots. By performing intersection analysis on these genes, we were able to obtain genes that showed high correlation (absolute correlation coefficient >0.4 and p-value <0.05) across multiple states, thereby providing a research foundation for subsequent model construction.

### 2.4 Machine learning based prognosis signature construction

To further evaluate the potential of epigenetic-related genes in PDAC prognosis prediction, this study employed a comprehensive and advanced set of machine learning methods. We integrated multiple algorithms, including Random Survival Forest (RSF) (Ishwaran et al., 2008), Elastic Net (Enet) (Zou and Hastie, 2005; Cho et al., 2009), Stepwise Cox Regression (StepCox) (Liu et al., 2023), CoxBoost (Binder and Binder, 2015), Partial Least Squares Cox Regression (plsRcox) (Bertrand et al., 2022; Bertrand et al., 2014), SuperPC (Bair et al., 2006), Gradient Boosting Machine (GBM) (Ayyadevara and Ayyadevara, 2018), Survival Support Vector Machine (survival-SVM) (Van Belle et al., 2011), Ridge Regression (Arashi et al., 2021), and Lasso Regression (Ranstam and Cook, 2018), to construct a series of prognostic prediction models. To ensure the reliability and generalization ability of the models, we used the TCGA dataset as the training set and selected two independent ICGC datasets as external validation sets. In the data preprocessing stage, we standardized all features to eliminate the influence of scale differences. Subsequently, we not only evaluated the performance of each algorithm individually but also explored up to 63 algorithm combinations, such as RSF + CoxBoost, Lasso + GBM, etc., aiming to obtain more stable and accurate prediction results. We used the C-index as the primary evaluation metric to comprehensively assess the discriminative ability of each model on both the training and validation sets, and presented the results in heatmap form for quick identification of the best models. For RSF, we used 1,000 trees and optimized the node size. Enet models were built with α values ranging from 0.1 to 0.9. CoxBoost models were optimized using cross-validation to determine the optimal number of boosting steps. For GBM, we used 10,000 trees with a maximum depth of 3 and a learning rate of 0.001. Lasso and Ridge regression models were fitted using 10-fold cross-validation to select the optimal λ value. Finally, we conducted in-depth analysis of the best-performing models, including feature importance ranking and result visualization, aiming to reveal the crucial role of epigenetic-related genes in PDAC prognosis and provide reliable data support for clinical individualized treatment decisions.

### 2.5 Relevant genes and risk score signature evaluation

For epigenetic-related genes, we conducted a series of network-based analyses. We performed univariate Cox regression analysis using the expression data of these genes, selecting genes significantly associated with patient survival (*p* < 0.05). We calculated their hazard ratios (HR) and p-values. Next, we computed the Spearman correlation coefficients (Pripp, 2018) between these genes and conducted correlation significance tests. Finally, we retained only gene pairs with absolute correlation coefficients greater than 0.4 and p-values less than 0.05 to ensure that the connections in the network have biological significance. Based on these data, we constructed a gene interaction network, where red represents positive correlations, blue represents negative correlations, and the thickness of the lines reflects the strength of the correlation. The igraph package was used for visualization (Csardi and Tamas, 2005).

Furthermore, to evaluate the predictive value of our developed epigenetic-related gene prognostic model in PDAC, we conducted comprehensive Cox regression analyses based on sample clinical characteristics. First, we performed univariate Cox regression analysis on clinical variables, including age, gender, T stage, N stage, M stage, clinical stage, and our risk score. After calculating the hazard ratio (HR), 95% confidence interval (CI), and p-value for each variable, we selected variables with statistical significance in the univariate analysis for multivariate Cox regression analysis. We used the forestplot package to create forest plots to display the HR and 95% CI for each variable.

### 2.6 Enrichment analysis

This study continued to employ various bioinformatics methods to explore the relationship between gene expression patterns and prognostic risk. We used the Gene Set Variation Analysis (GSVA) method (Hänzelmann et al., 2013), utilizing the GSVA package to perform ssGSEA on the PDAC dataset, quantifying the activity of specific pathways in each sample. We chose the Hallmark gene set (Liberzon et al., 2015) as a reference, ensuring biological relevance of the analysis. Subsequently, we compared the pathway activity differences between high-risk and low-risk groups, using the limma package for differential analysis (Ritchie et al., 2015). To visually present the results, we generated a bar plot showing significantly different pathways and their t-values. Additionally, we calculated the correlation between GSVA scores and risk scores, visualizing these relationships through a heatmap. For pathways with significant statistical differences, we used Cox proportional hazards regression analysis to quantify the association strength between pathway activity and survival risk.

### 2.7 Mutation analysis

To further explore the association between the gene risk model and tumor mutation characteristics, we conducted a comprehensive mutation analysis on the PDAC dataset. First, we calculated the tumor heterogeneity (MATH) score for each sample using the maftools package (Mayakonda et al., 2018), and compared the differences between high and low-risk groups. Subsequently, we divided the samples into high and low-risk groups, generating mutation landscape plots (oncoplots) for each group, showing the 20 most common mutated genes and their frequencies. To understand the mutation patterns more deeply, we also performed somatic mutation interaction analysis, revealing the co-occurrence and mutual exclusivity of gene mutations in high and low-risk groups. This is significant for revealing and understanding the potential link between risk scores and tumor mutation burden and heterogeneity.

### 2.8 Immune analysis

The epigenetic-related gene risk model is closely related to the tumor immune microenvironment. We used the IOBR package (Zeng et al., 2021) to assess ESTIMATE, CIBERSORT, and immune cell subpopulation infiltration in PDAC samples. First, we used the ESTIMATE algorithm to calculate stromal scores, immune scores, and ESTIMATE scores for each sample, and compared the differences between high and low-risk groups. Subsequently, we used the ssGSEA method to perform enrichment analysis on immune-related pathways, and visualized the significantly different pathway activities between high and low-risk groups through heatmaps. To understand the composition of tumor immune cells in more detail, we used the CIBERSORT algorithm to perform deconvolution analysis on 22 immune cell subpopulations (Chen et al., 2018). Through violin plots, we visually demonstrated the differences in immune cell composition between high and low-risk groups. Additionally, we calculated the Spearman correlation between these immune cell subpopulations and risk scores, and used bubble plots to show the correlation strength and statistical significance.

### 2.9 Drug sensitivity analysis

The Genomics of Drug Sensitivity in Cancer (GDSC) database (https://www.cancerrxgene.org/) is commonly used to predict tumor sensitivity to drugs (Yang et al., 2013). In this study, we utilized the GDSC database and the pRRophetic package (Geeleher et al., 2014) to explore the potential application of our epigenetic-related gene risk model in drug sensitivity prediction. We first obtained drug sensitivity data and gene expression data from the GDSC database, then used the pRRopheticPredict function to predict drug sensitivity for each sample in the TCGA-PDAC dataset. Based on some clinical drugs in the GDSC database, we predicted and compared drug sensitivity differences between high and low-risk groups. For each drug, we used the Wilcoxon rank-sum test to compare the differences in predicted IC50 values between high and low-risk groups, and created box plots for visualization (Sebaugh, 2011). These results not only revealed potential connections between our risk model and drug responses but also provided new insights for personalized treatment of PDAC.

### 2.10 Pathological validation

To validate the biological significance of our model and examine the gene expression trends, we conducted further pathological verification on the top five genes with the highest weights in our model. On one hand, we utilized the Human Protein Atlas (HPA) database (https://www.proteinatlas.org/) to compare the protein expression levels of these genes in pancreatic cancer tissues and adjacent relatively normal pancreatic tissues (Uhlén et al., 2015). For genes lacking data in the HPA database, we performed immunohistochemical staining verification in our laboratory. All patients provided written informed consent, and this research protocol was approved by the Medical Ethics Committee of the Affiliated Hospital of Qingdao University (approval number: QYFYWZLL28682).

Besides, gene expression was further validated by qRT-PCR. PANC-1 (pancreatic cancer) and hTERT-HPNE (normal pancreatic ductal) cell lines were cultured in DMEM supplemented with 10% FBS and 1% penicillin/streptomycin under standard conditions (37°C, 5% CO2). Total RNA was extracted using RNAios Plus reagent following the manufacturer’s protocol. RNA was reverse transcribed using ABScript III RT Master Mix, and qRT-PCR was performed using Universal SYBR Green Fast qPCR Mix. The sequences of primers used for qRT-PCR are listed in Table 1. Relative gene expression was calculated using the 2^−ΔΔCT^ method.

**TABLE 1:** Primer sequences for qRT-PCR.

Gene	Direction	Primer sequence (5′-3′)
KRTCAP2	Forward	CTC​TTC​GTG​TTC​TCG​CTC​ACT
	Reverse	CAG​GTG​GTG​ACA​CAG​ACT​CG
NENF	Forward	AGA​TCA​GCC​CAT​CTA​CTT​GGC
	Reverse	CTT​CCC​CGT​CAA​GGC​ATT​G
PSAP	Forward	CCC​GGT​CCT​TGG​ACT​GAA​AG
	Reverse	TAT​GTC​GCA​GGG​AAG​GGA​TTT
MRPL41	Forward	GTT​CGT​CGT​CCC​GGA​TCT​G
	Reverse	GTA​GCT​CAC​GTA​GGG​CTT​GA
S100A16	Forward	ATG​TCA​GAC​TGC​TAC​ACG​GAG
	Reverse	GTT​CTT​GAC​CAG​GCT​GTA​CTT​AG

## 3 Results

### 3.1 Single-cell RNA sequencing reveals cellular heterogeneity and epigenetic characteristics in the PDAC microenvironment

**FIGURE 2:**
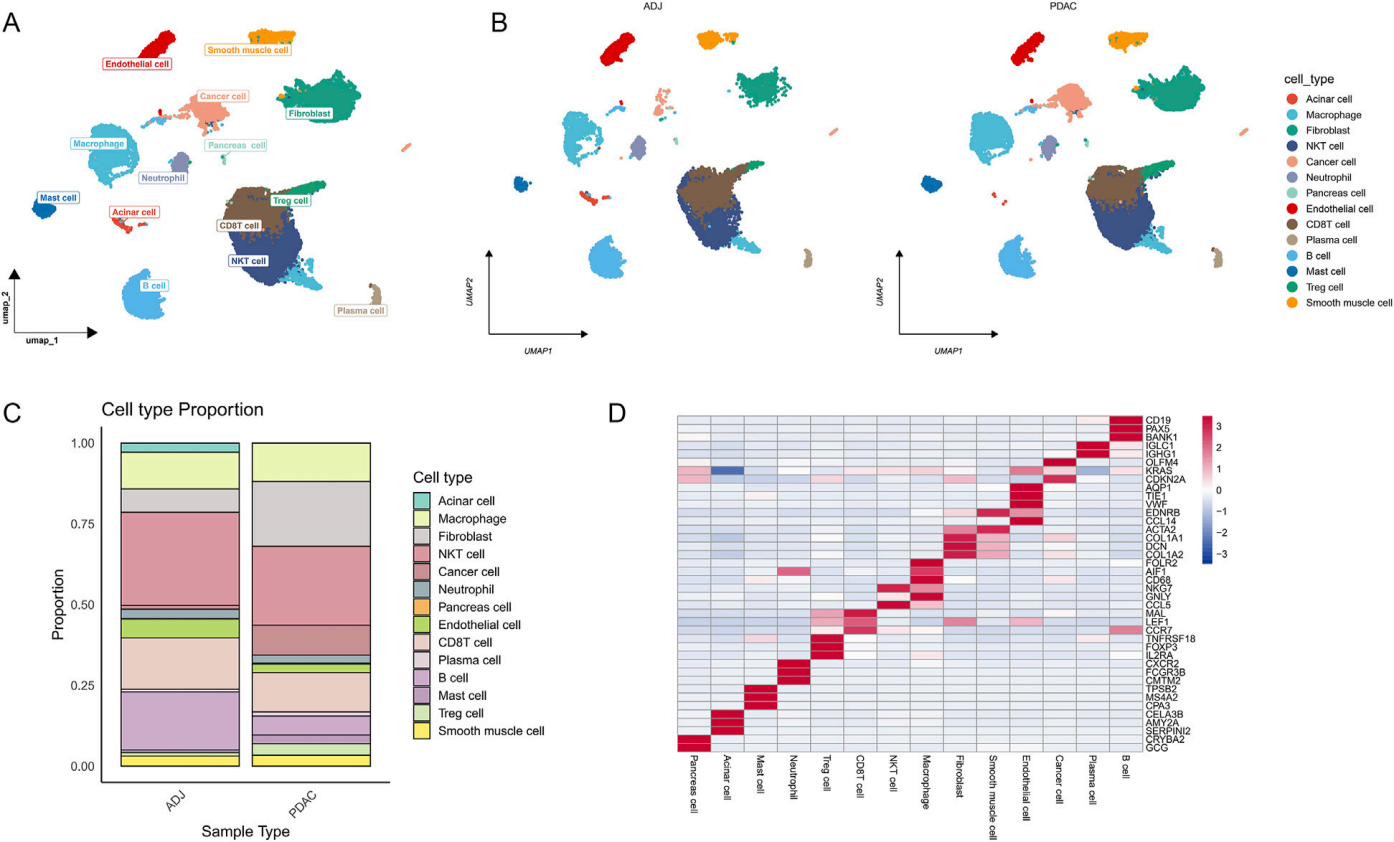
Single-cell RNA sequencing reveals cellular heterogeneity in PDAC and its adjacent tissues. **(A)** UMAP plot of single-cell transcriptomes from PDAC and adjacent tissue samples, showing 14 major cell types. **(B)** UMAP plot of cell type distribution in PDAC and ADJ samples. **(C)** Stacked bar plot showing the proportion of each cell type in PDAC and ADJ samples, demonstrating tumor microenvironment remodeling. **(D)** Heatmap of marker gene expression for each cell cluster, validating the accuracy of cell type annotation.

We performed single-cell RNA sequencing analysis on 6 PDAC adjacent normal tissue samples (ADJ) and 6 PDAC samples from the GSE212966 dataset. Fourteen major cell types were identified and annotated (Figure 2A). These cell types include cancer cells, fibroblasts, endothelial cells, smooth muscle cells, and various immune cell subpopulations such as macrophages, T cells, and B cells.

Comparison of cell composition between ADJ and PDAC samples (Figure 2B) revealed significant microenvironment remodeling in PDAC tissues. The proportions of cancer cells, fibroblasts, mast cells, and Treg cells were markedly increased in PDAC samples, while the proportions of endothelial cells and B cells substantially decreased (Figure 2C). To further validate the accuracy of cell type annotation, we plotted a heatmap showing the expression of marker genes for each cell cluster (Figure 2D).

### 3.2 Single-cell level histone modification characteristics in PDAC

**FIGURE 3:**
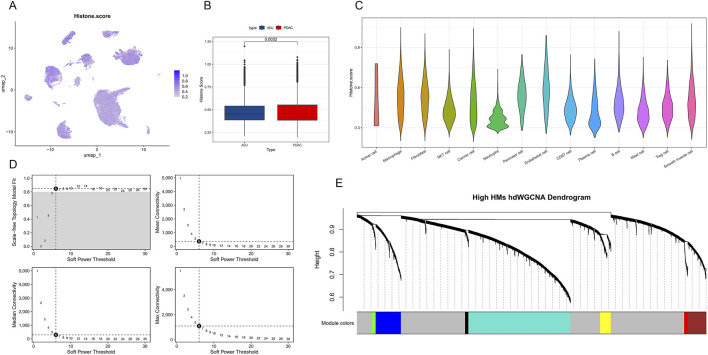
Single-cell level histone modification characteristics and co-expression network analysis in PDAC. **(A)** Single-cell UMAP plot showing the distribution of histone modification scores (Histone score). Color depth represents score levels. **(B)** Box plot comparing histone modification scores between PDAC and ADJ samples. **(C)** Violin plot showing the distribution of histone modification scores across different cell types in PDAC. **(D)** Soft threshold parameter selection plot for hdWGCNA network construction. Includes curves of scale-free topology fit index R^2^ and mean connectivity versus soft threshold. **(E)** Hierarchical clustering dendrogram of gene co-expression modules under high histone modification states. Different colors represent different co-expression modules.

To investigate the role of epigenetic regulation in PDAC in depth, we conducted a detailed analysis of the expression patterns of histone modification-related genes. Using the ssGSEA method, we calculated a histone modification score (Histone score) for each single cell. Figure 3A shows the distribution of histone modification scores on the UMAP plot. Further comparison revealed that cells in PDAC samples generally had higher histone modification scores, showing significant differences compared to ADJ samples (p = 0.0032, Figure 3B). This suggests that PDAC cells may enhance histone modifications to regulate gene expression, thereby promoting tumor progression. Cell type-specific analysis showed no significant differences in histone modification levels among different cell populations in PDAC (Figure 3C).

To further reveal the co-expression patterns of histone modification-related genes, we applied the hdWGCNA method to construct a weighted gene co-expression network. By analyzing soft threshold parameters (Figure 3D), we determined the optimal network construction parameters. The final hierarchical clustering dendrogram (Figure 3E) shows the gene module structure under high histone modification states, with different colors representing different co-expression modules. These modules may reflect functionally coordinated gene sets during PDAC progression.

### 3.3 Single-cell level DNA methylation characteristics in PDAC

**FIGURE 4:**
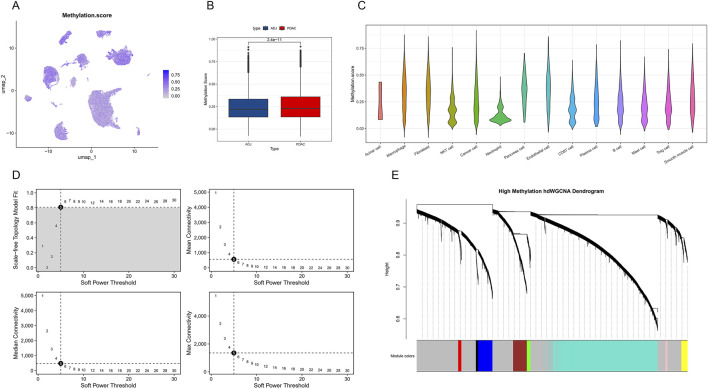
Single-cell level DNA methylation characteristics and co-expression network analysis in PDAC. **(A)** Single-cell UMAP plot showing the distribution of DNA methylation scores (Methylation score). **(B)** Box plot comparing DNA methylation scores between PDAC and ADJ samples. **(C)** Violin plot showing the distribution of DNA methylation scores across different cell types in PDAC. **(D)** Soft threshold parameter selection plot for hdWGCNA network construction. Includes curves of scale-free topology fit index R^2^ and mean connectivity versus soft threshold. **(E)** Hierarchical clustering dendrogram of gene co-expression modules under high DNA methylation states.

Similar to the previous section, we further analyzed the expression patterns of DNA methylation-related genes in PDAC. By calculating a DNA methylation score (Methylation score) for each single cell and displaying it on a UMAP plot (Figure 4A), we found that cells in PDAC samples generally have higher DNA methylation scores, showing highly significant differences compared to ADJ samples (p = 2.4e-11, Figure 4B). This finding suggests that PDAC cells may enhance DNA methylation to regulate gene expression. Cell type-specific analysis revealed no significant differences in DNA methylation levels among different cell populations in PDAC (Figure 4C). Subsequently, we applied the hdWGCNA method to construct a weighted gene co-expression network. By analyzing soft threshold parameters (Figure 4D), we determined the optimal network construction parameters. The final hierarchical clustering dendrogram (Figure 4E) shows the gene module structure under high DNA methylation states.

### 3.4 WGCNA reveals key gene modules of epigenetic regulation in PDAC

To further explore the role of epigenetic regulation in PDAC, we performed WGCNA on the TCGA-PDAC dataset. We analyzed histone modification and DNA methylation-related genes separately.

**FIGURE 5:**
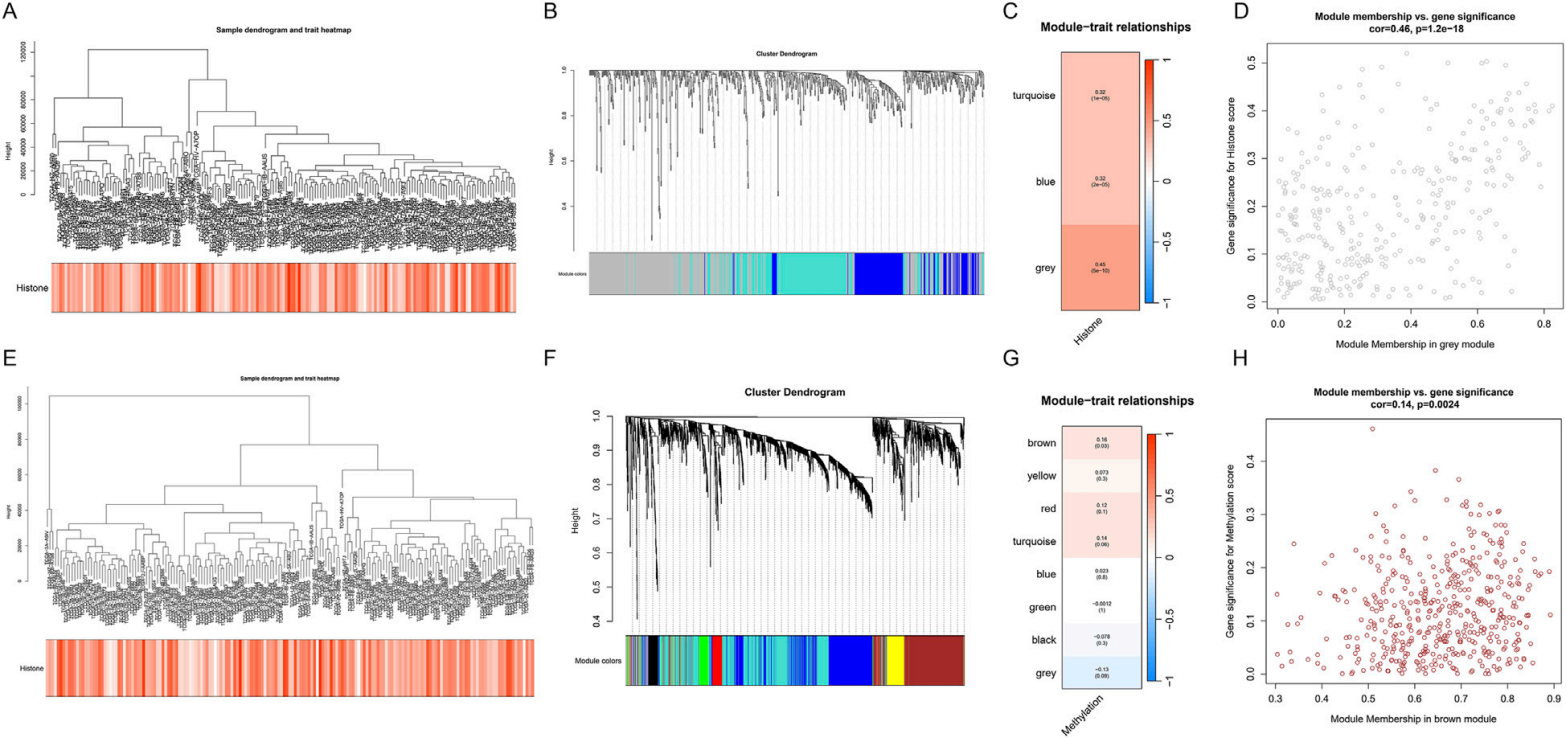
WGCNA analysis of TCGA-PDAC dataset reveals key gene modules related to epigenetic regulation. **(A–D)** WGCNA analysis of histone modification-related genes. **(A)** Sample clustering dendrogram and histone modification score heatmap. **(B)** Gene dendrogram and module assignment. **(C)** Module-trait relationship heatmap. **(D)** Module membership vs. gene significance scatter plot for the grey module. **(E–H)** WGCNA analysis of DNA methylation-related genes. **(E)** Sample clustering dendrogram and DNA methylation score heatmap. **(F)** Gene dendrogram and module assignment. **(G)** Module-trait relationship heatmap. **(H)** Module membership vs. gene significance scatter plot for the brown module.

The WGCNA results for histone modification-related genes are shown in Figures 5A–D. Sample clustering and trait heatmap (Figure 5A) display sample similarity and the distribution of histone modification scores. The gene dendrogram and module assignment (Figure 5B) reveal three main co-expression modules. Module-trait relationship analysis (Figure 5C) indicates that the grey module shows the strongest positive correlation with histone modification scores (correlation coefficient = 0.45, p = 1e-09). Further module membership vs. gene significance scatter plot (Figure 5D) shows that genes in the grey module have high module membership and gene significance.

Similarly, WGCNA results for DNA methylation-related genes are shown in Figures 5E–H. Module-trait relationship analysis (Figure 5G) shows that the brown module has the strongest positive correlation with DNA methylation scores (correlation coefficient = 0.55, p = 2e-15). The module membership vs. gene significance scatter plot (Figure 5H) further confirms the importance of genes in the brown module.

### 3.5 Integrated analysis reveals key genes and pathways of epigenetic regulation in PDAC

**FIGURE 6:**
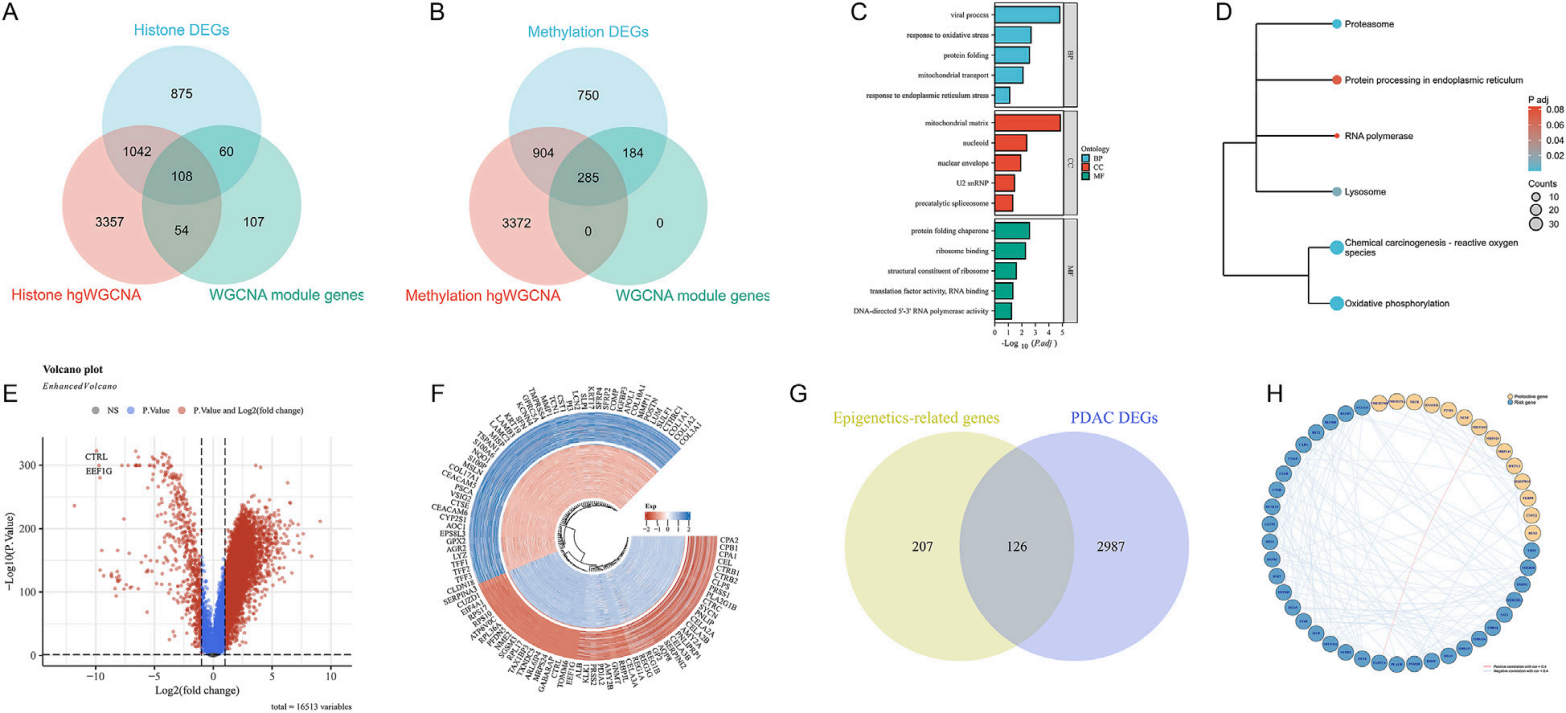
Integrated analysis of key genes in epigenetic regulation in PDAC. **(A)** Venn diagram of histone modification-related genes in different analysis methods. **(B)** Venn diagram of DNA methylation-related genes in different analysis methods. **(C)** GO functional enrichment analysis results of epigenetic key genes. **(D)** KEGG pathway enrichment analysis results of epigenetic key genes. **(E)** Volcano plot of differentially expressed genes between normal samples and PDAC. **(F)** Circular plot of top 50 PDAC DEGs. **(G)** Venn diagram showing overlap between epigenetic-related genes and PDAC differentially expressed genes. **(H)** Protein-protein interaction network of key genes.

We found that 108 genes were simultaneously identified in differential expression analysis, hdWGCNA, and WGCNA for histone modifications (Figure 6A). In DNA methylation-related analysis, 285 genes were commonly identified (Figure 6B). Functional enrichment analysis revealed the biological processes and molecular functions involved in these epigenetic key genes (Figure 6C). Among them, protein folding and mitochondrial function processes were significantly enriched. KEGG pathway analysis further identified several signaling pathways closely related to PDAC, including Chemical carcinogenesis - reactive oxygen species, Oxidative phosphorylation, etc. (Figure 6D). Differential expression analysis results between normal samples and PDAC are shown in a volcano plot (Figure 6E), where red and blue dots represent upregulated and downregulated genes, respectively. The circular plot (Figure 6F) visually displays the top 50 PDAC DEGs with the most significant expression changes. Furthermore, we compared the overlap between epigenetic-related genes and PDAC DEGs (Figure 6G). The results show that 126 genes are both related to epigenetic regulation and differentially expressed in PDAC. Finally, we constructed a protein-protein interaction network of these key genes (Figure 6H), showing a high positive correlation between NDUFA13 in protective genes and PABPC4 in risk genes.

### 3.6 Machine learning models for predicting PDAC patient prognosis

**FIGURE 7:**
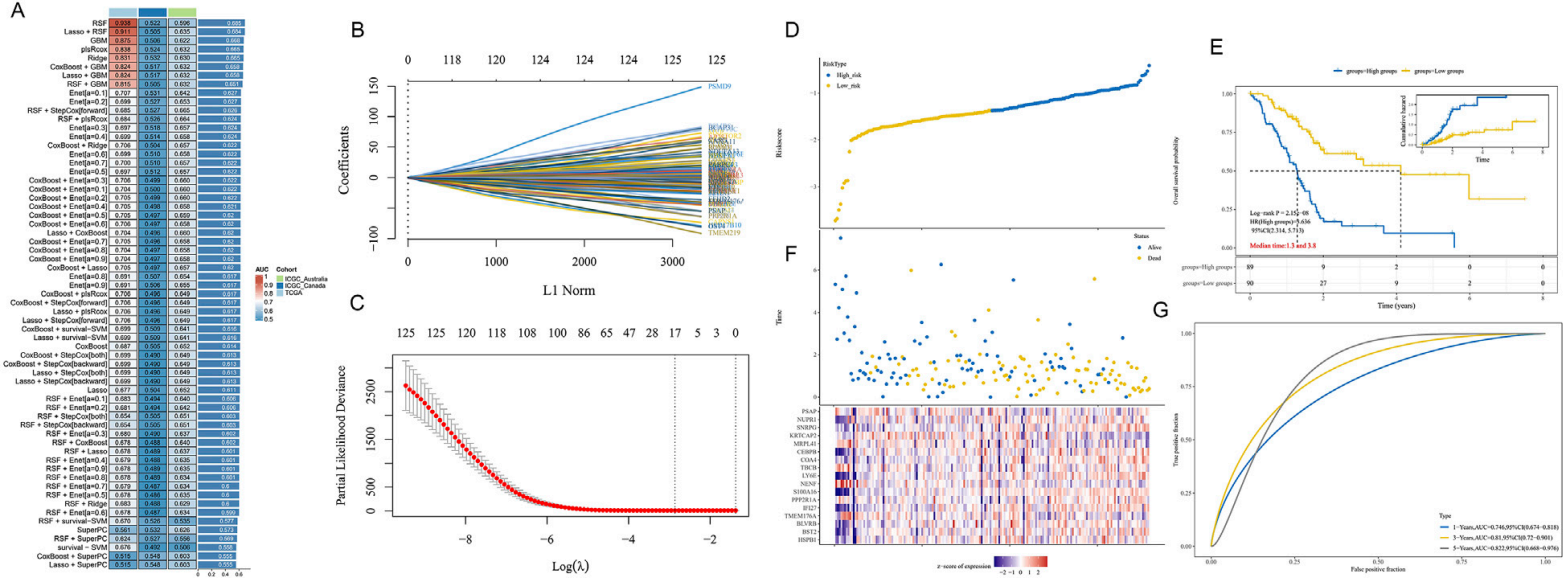
PDAC prognostic prediction model based on epigenetic-related genes. **(A)** Performance comparison heatmap of different machine learning algorithm combinations. **(B)** L1 regularization path diagram of Lasso regression, showing the feature selection process. **(C)** Trend graph of Lasso regression coefficients changing with penalty coefficient λ. **(D)** Patient risk score distribution plot based on selected features. **(E)** Kaplan-Meier survival curves for high-risk and low-risk groups. **(F)** Expression heatmap of key prognostic-related genes in the model for high-risk and low-risk groups. **(G)** Time-dependent ROC curve analysis of the prognostic model.

To assess the potential of epigenetic-related genes in predicting PDAC prognosis, we applied various machine learning algorithms to build prognostic models. Figure 7A shows a performance comparison of different algorithm combinations, with the Lasso + RSF combination performing best across multiple evaluation metrics. Figures 7B, C illustrate the feature selection process of Lasso regression. Through L1 regularization (Figure 7B), we gradually increased the penalty coefficient λ to select the optimal feature subset. Figure 7C’s partial least squares path graph shows the change trend of different feature coefficients as λ increases, helping us identify the most stable and important prognostic-related genes. The final weight calculation formula is as follows: Riskscore = (0.005)*HSPB1 + (0.0111)*BST2 + (0.0176)*BLVRB + (−0.0262)*TMEM176A + (0.0054)*IFI27 + (−0.0408)*PPP2R1A + (0.1781)*S100A16 + (−0.2303)*NENF + (0.0952)*LY6E + (−0.0303)*TBCB + (0.1254)*COA4 + (0.0909)*CEBPB + (−0.1997)*MRPL41 + (−0.2693)*KRTCAP2 + (0.1462)*SNRPG + (0.0278)*NUPR1 + (−0.2068)*PSAP.

Based on the selected features, we calculated a risk score for each patient and divided patients into high-risk and low-risk groups. Figure 7D clearly shows the separation of the two patient groups in the risk score distribution plot. Figure 7E’s Kaplan-Meier survival curve further confirms the significant difference in survival time between high-risk and low-risk groups (p < 0.001). Figure 7F’s heatmap shows the expression patterns of key genes in the model for high-risk and low-risk groups. We observed clear differential expression of these genes between the two groups, further supporting the rationality of our risk score model. Finally, Figure 7G’s time-dependent ROC curve analysis evaluated the predictive accuracy of our model. The results show that the model demonstrates good discriminative ability in predicting 1-year, 3-year, and 5-year prognosis, with AUC values of 0.746, 0.81, and 0.822, respectively.

These results suggest that machine learning models based on epigenetic-related genes can effectively predict the prognosis of PDAC patients, providing a potential tool for individualized treatment decisions.

### 3.7 The role of gene expression features and clinical factors in PDAC prognosis

**FIGURE 8:**
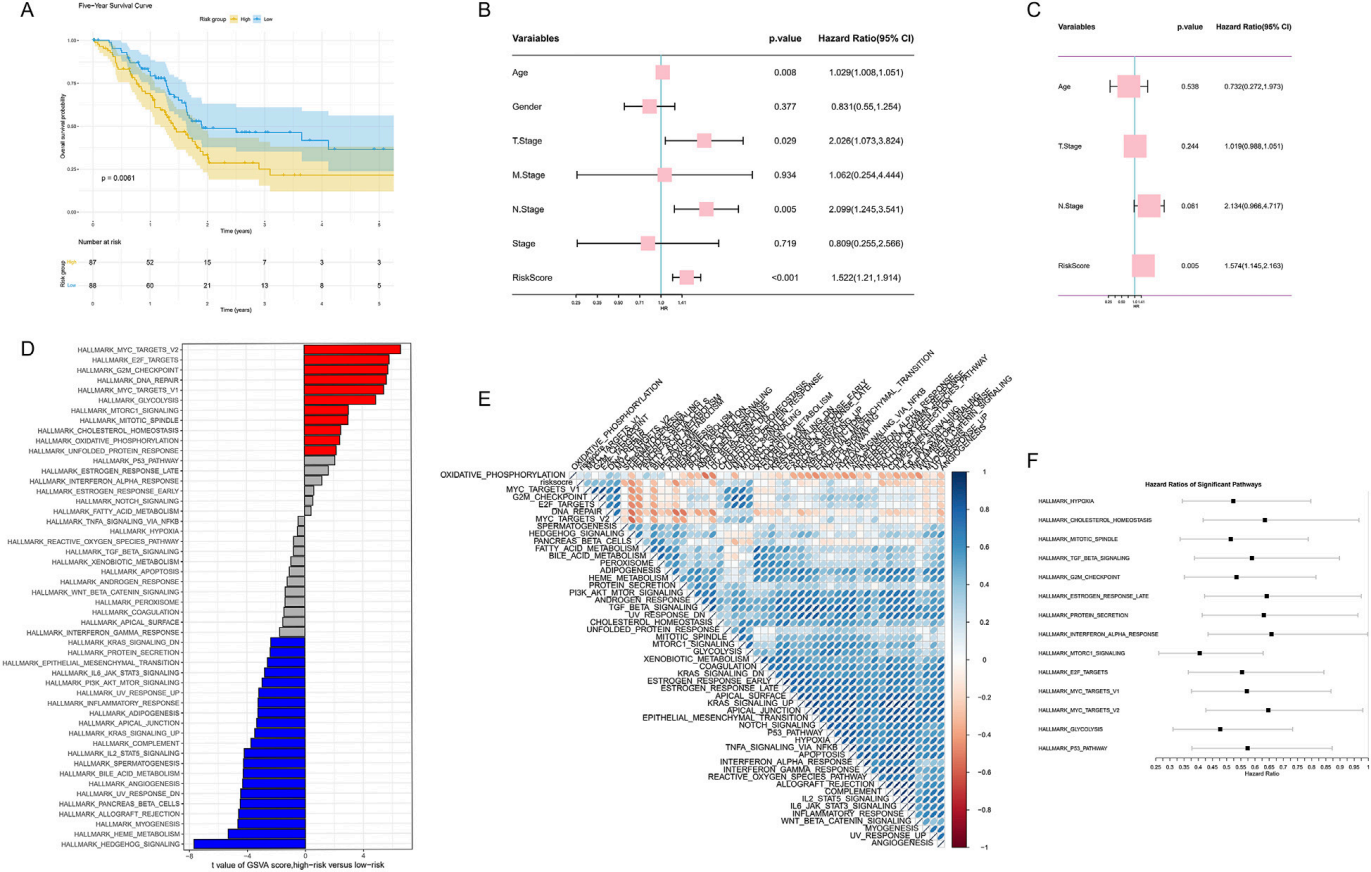
Comprehensive analysis of gene expression features and clinical factors in PDAC prognosis. **(A)** Kaplan-Meier survival curve based on risk scores. **(B)** Forest plot of univariate Cox regression analysis. **(C)** Forest plot of multivariate Cox regression analysis. **(D)** Bar plot of GSVA score differences between high-risk and low-risk groups. **(E)** Correlation heatmap of significantly different pathways. **(F)** HR forest plot of major pathways based on GSVA analysis.

To further validate our prognostic model and explore the role of gene expression features and clinical factors in PDAC prognosis, we conducted a series of analyses. Based on our risk score model, we plotted Kaplan-Meier survival curves, which showed that high-risk group patients had significantly lower survival rates than the low-risk group (p = 0.0061, Figure 8A). We performed univariate Cox regression analysis, revealing multiple factors associated with prognosis, including age, T stage, N stage, and our risk score (Figure 8B). Multivariate Cox regression analysis further confirmed that our risk score is an independent prognostic indicator beyond other clinical factors (p = 0.005, Figure 8C).

To gain deeper insights into molecular differences between high-risk and low-risk groups, we conducted GSVA analysis. The results showed significant differences in multiple pathways between high-risk and low-risk groups, with pathways such as MYC V2 targets, E2F targets, G2M checkpoint, and DNA repair enriched in the high-risk group (Figure 8D). We further constructed a correlation heatmap of these significant pathways, revealing potential functional connections between them (Figure 8E). Finally, we plotted a forest plot of Hazard Ratios for major pathways based on GSVA analysis, further highlighting the importance of these pathways in PDAC prognosis (Figure 8F).

### 3.8 Mutation characteristic analysis of epigenetic regulation genes in PDAC

**FIGURE 9:**
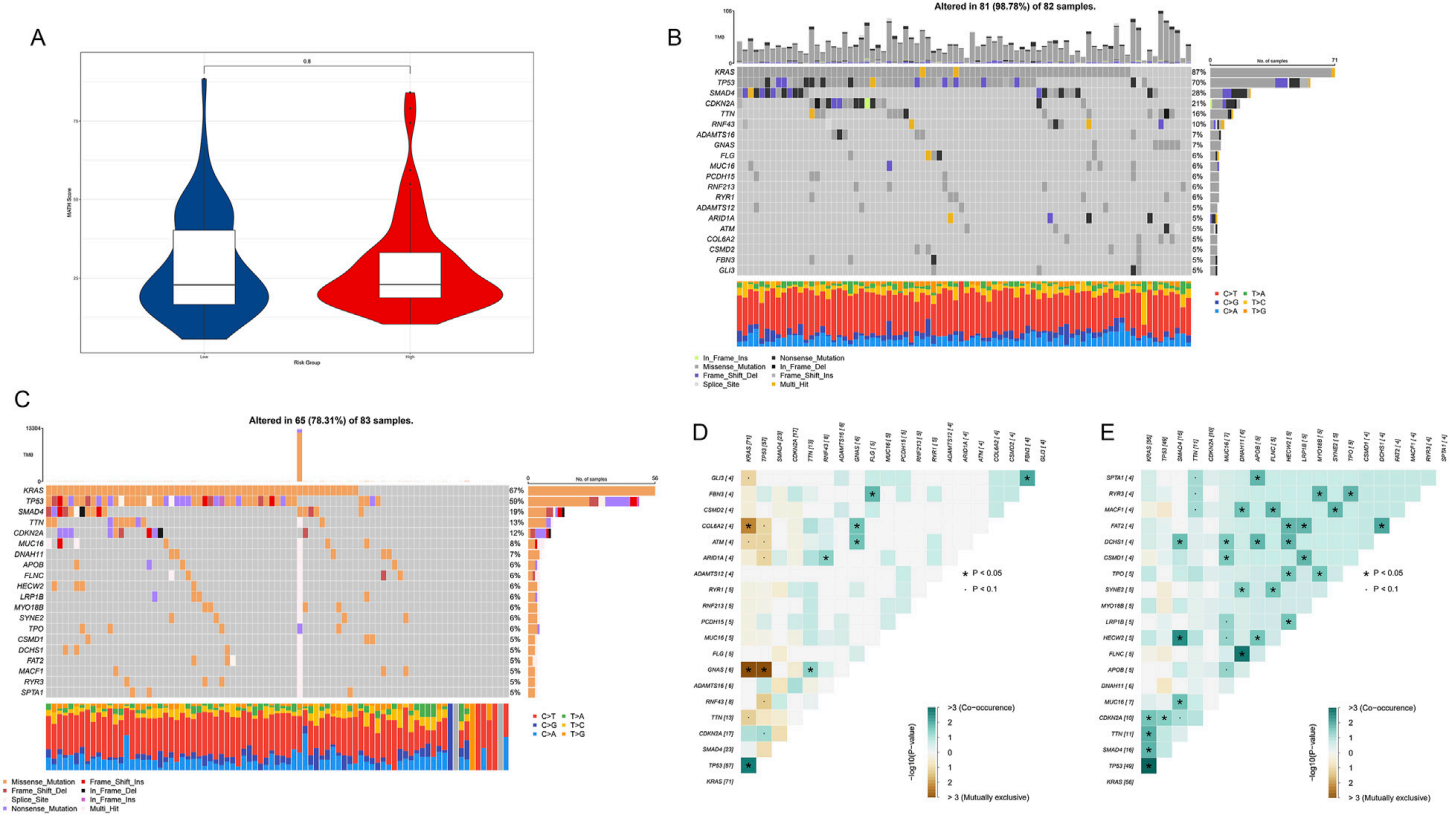
Mutation characteristic analysis of epigenetic regulation genes in PDAC. **(A)** Violin plot comparing mutation burden between high-risk and low-risk patients. **(B)** Mutation frequency and types of major genes in 82 high-risk samples. **(C)** Mutation frequency and types of major genes in 83 low-risk samples. **(D)** Co-occurrence and mutual exclusivity relationship heatmap of major mutated genes in the high-risk group. **(E)** Co-occurrence and mutual exclusivity relationship heatmap of major mutated genes in the low-risk group.

To investigate the mutation characteristics of epigenetic regulation genes in PDAC, we conducted a detailed analysis of genomic data from high-risk and low-risk patients. First, we compared the mutation burden between high-risk and low-risk patients. The results showed no significant difference in mutation burden between high-risk and low-risk groups (p = 0.8, Figure 9A).

We further analyzed frequently mutated genes in high-risk and low-risk groups. The results showed that in 82 high-risk samples, 81 (98.78%) had at least one mutation in the analyzed genes (Figure 9B). Among them, KRAS, TP53, and SMAD4 were the three genes with the highest mutation frequencies, occurring in 87%, 70%, and 28% of samples, respectively. In 83 low-risk samples, 65 (78.31%) had at least one mutation in the analyzed genes (Figure 9C). The genes with the highest mutation frequencies were highly consistent with previous analysis results, further confirming the importance of these genes in PDAC development.

We observed some significant gene mutation co-occurrence and mutual exclusivity patterns in both high-risk (Figure 9D) and low-risk groups (Figure 9E). In the high-risk group, KRAS and TP53 frequently co-occurred, while GNAS rarely appeared simultaneously with KRAS and TP53 in the same patient. In low-risk patients, the co-occurrence of KRAS and TP53 remained strong, while more genes showed co-occurrence patterns, and there were no obvious mutual exclusivity patterns.

### 3.9 Association analysis of epigenetic regulation and immune microenvironment in PDAC

**FIGURE 10:**
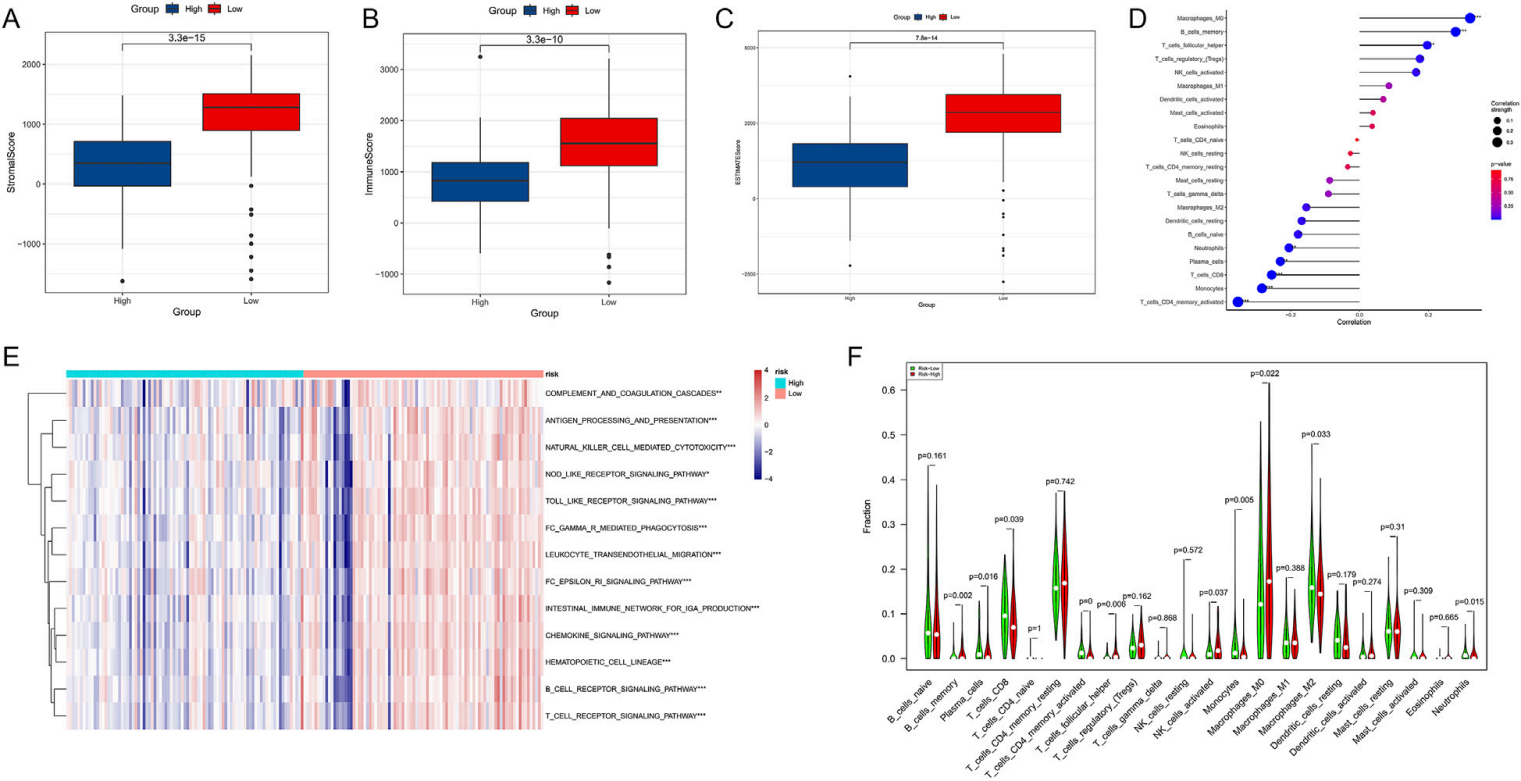
Association analysis of epigenetic regulation and immune microenvironment in PDAC. **(A)** Comparison of Stromal Score between high-risk and low-risk patients. **(B)** Comparison of Immune Score between high-risk and low-risk patients. **(C)** Comparison of ESTIMATE Score between high-risk and low-risk patients. **(D)** Correlation analysis between different immune cell types and risk scores. **(E)** Activity heatmap of immune-related pathways in high-risk and low-risk groups. **(F)** Comparison of various immune cell subset proportions between high-risk and low-risk patients.

To explore the relationship between epigenetic regulation and the immune microenvironment in PDAC, we conducted a series of immune-related analyses on high-risk and low-risk patients. First, we compared the Stromal Score, Immune Score, and ESTIMATE Score between the two groups. Results showed that low-risk patients had significantly higher Stromal Score (Figure 10A, *p* = 3.3e-15), Immune Score (Figure 10B, p = 3.3e-10), and ESTIMATE Score (Figure 10C, p = 7.8e-14) than high-risk patients, suggesting that low-risk patients may have richer tumor stromal components and immune cell infiltration.

Correlation analysis (Figure 10D) revealed significant associations between various immune cells and risk scores. Among them, M0 macrophages, memory B cells, follicular helper T cells, and Treg cells showed positive correlations with high risk scores, while CD4^+^ active memory T cells, monocytes, and CD8^+^ T cells showed positive correlations with low risk scores. The heatmap (Figure 10E) displayed differences in the activity of multiple immune-related pathways between high-risk and low-risk groups. The low-risk group generally showed higher immune pathway activity, consistent with previous immune score results. Finally, we compared the proportions of various immune cell subsets between the two groups (Figure 10F). Results showed significant differences in multiple immune cell subsets between high-risk and low-risk groups. For example, the proportions of CD8^+^ T cells, monocytes, and M2 macrophages were significantly higher in the low-risk group, while the proportion of M0 macrophages was significantly increased in the high-risk group.

### 3.10 Drug sensitivity analysis of high-risk and low-risk PDAC patients

To investigate the potential impact of epigenetic regulation patterns on drug responses in PDAC patients, we conducted a series of drug sensitivity analyses on high-risk and low-risk patients. We selected multiple drugs commonly used in PDAC treatment or clinical trials for evaluation.

**FIGURE 11:**
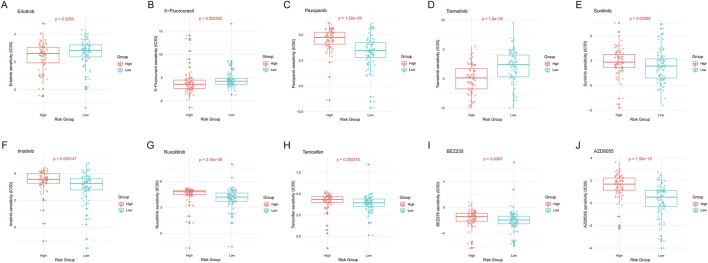
Drug sensitivity analysis of high-risk and low-risk PDAC patients. **(A)** Erlotinib. **(B)** 5-Fluorouracil. **(C)** Pazopanib. **(D)** Trametinib. **(E)** Sunitinib. **(F)** Imatinib. **(G)** Ruxolitinib. **(H)** Tamoxifen. **(I)** BEZ235. **(J)** AZD8055. Each subplot shows the distribution of IC50 values for the respective drug in high- and low-risk groups, with p-values indicating the statistical significance of the difference between groups.

Erlotinib and Trametinib are EGFR and MEK inhibitors, respectively. Results showed that low-risk patients had significantly higher sensitivity to these drugs compared to high-risk patients (p = 0.0259, Figure 11A; p = 1.9e-06, Figure 11D). 5-Fluorouracil is a commonly used chemotherapy drug, and we similarly found that low-risk patients had significantly higher sensitivity to this drug (p = 0.000392, Figure 11B). This suggests that low-risk patients may be more suitable for EGFR and MEK targeted therapies, as well as 5-FU-based chemotherapy regimens.

Pazopanib and Sunitinib are both multi-target tyrosine kinase inhibitors. Analysis results showed that high-risk patients had significantly higher sensitivity to these drugs compared to low-risk patients (p = 1.32e-09, Figure 11C; p = 0.00385, Figure 11E). Imatinib, Ruxolitinib, Tamoxifen, BEZ235, and AZD8055 also showed similar differential patterns (Figures 11F–J). This indicates that these drugs may be more suitable for high-risk PDAC patients.

Overall, these drug sensitivity analysis results emphasize the close association between epigenetic regulation patterns and drug responses in PDAC patients, providing new perspectives and potential strategies for precision treatment of PDAC.

### 3.11 Pathological validation

**FIGURE 12:**
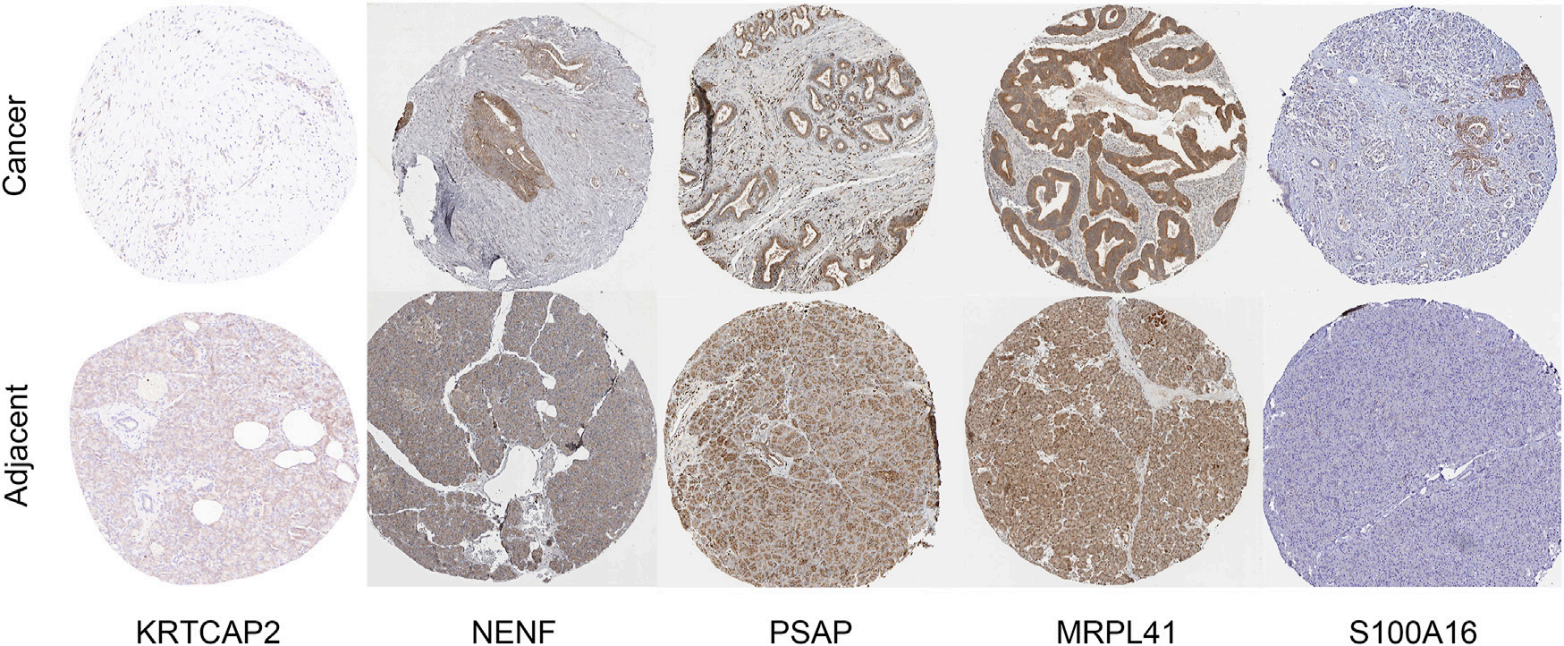
Immunohistochemical staining of KRTCAP2, NENF, PSAP, MRPL41, and S100A16. Representative immunohistochemical staining images showing the protein expression of KRTCAP2, NENF, PSAP, MRPL41, and S100A16 in PDAC tissues and adjacent normal pancreatic tissues. Images were obtained from both our hospital cohort and the HPA database.

We performed immunohistochemistry validation for the top five weighted genes: KRTCAP2, NENF, PSAP, MRPL41, and S100A16. For KRTCAP2, we used patient samples from the hospital. For the remaining 4 genes, we cited results from the HPA database. In Figure 12, compared to adjacent tissue, KRTCAP2 are significantly downregulated in PDAC tissues, NENF, PSAP, MRPL41, and S100A16 are significantly upregulated in PDAC tissues.

**FIGURE 13:**
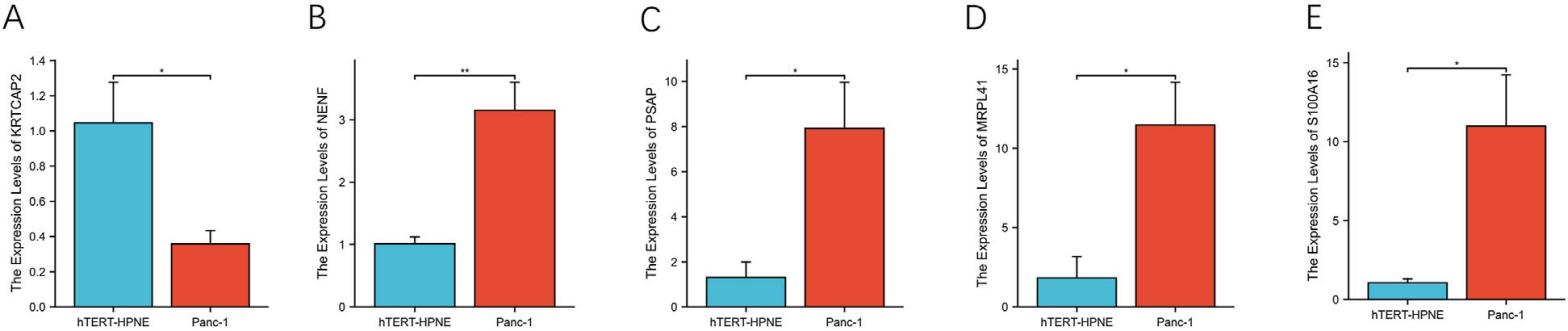
Differential expression analysis of prognostic genes in pancreatic cell lines **(A–E)**. The relative mRNA expression levels of KRTCAP2, NENF, PSAP, MRPL41, and S100A16 were quantified by qRT-PCR in pancreatic cancer cell line PANC-1 and normal pancreatic epithelial cell line hTERT-HPNE.

To further validate the expression patterns observed in tissue samples, we examined the mRNA levels of these genes in PDAC cell line PANC-1 and normal pancreatic ductal cell line hTERT-HPNE using qRT-PCR (Figure 13). Consistent with the tissue results, KRTCAP2 showed significantly lower expression in PANC-1 cells compared to hTERT-HPNE cells (Figure 13A, p < 0.01). Conversely, the expression levels of NENF, PSAP, MRPL41, and S100A16 were significantly higher in PANC-1 cells than in hTERT-HPNE cells (Figures 13B–E, all p < 0.01). These findings in cell lines were in agreement with our observations in clinical specimens, further confirming the differential expression patterns of these genes in PDAC.

## 4 Discussion

This study comprehensively investigated the role and clinical significance of epigenetic regulation in PDAC by integrating single-cell RNA sequencing, epigenetic analysis, and machine learning methods (Wang Q. et al., 2023; Li et al., 2024). We first performed single-cell RNA sequencing analysis on PDAC and adjacent normal tissues, revealing cellular heterogeneity and compositional changes in the tumor microenvironment. Subsequently, we analyzed histone modifications and DNA methylation characteristics at the single-cell level, discovering that cells in PDAC samples generally exhibited higher levels of epigenetic modifications. Through WGCNA, we identified key gene modules highly correlated with histone modifications and DNA methylation, and conducted functional enrichment analysis. Based on key epigenetic-related genes, we constructed a machine learning model for PDAC prognosis prediction. Additionally, we analyzed the associations between epigenetic features and gene mutation patterns, immune microenvironment, and drug sensitivity.

Overall, the findings of this study not only deepen our understanding of PDAC molecular mechanisms but also provide new perspectives for PDAC diagnosis, prognosis assessment, and treatment. Epigenetic regulation plays a central role in the occurrence and development of PDAC, influencing multiple stages from tumor initiation to progression (Montalvo-Javé et al., 2023). Our research reveals that PDAC cells generally exhibit high levels of histone modifications and DNA methylation activity, which may confer stronger adaptability and survival advantages to tumor cells. Meanwhile, abnormal epigenetic regulation may also be one of the important factors driving PDAC tumor heterogeneity (Espinet et al., 2022). Different subclones may maintain their unique phenotypic and functional characteristics through specific epigenetic regulatory patterns, thereby promoting the overall adaptability of the tumor (Orlacchio et al., 2024). Furthermore, our analysis revealed significant immune microenvironment differences between risk groups, with the low-risk group showing higher immune and stromal scores, particularly increased infiltration of CD8^+^ T cells and M2 macrophages, suggesting stronger anti-tumor immune responses. These findings have crucial clinical implications, providing not only a theoretical basis for differential immunotherapy responses but also new evidence for immunotherapy strategy selection. For instance, high-risk patients might benefit from additional immune modulatory treatments, particularly immune checkpoint inhibitors, given their higher proportions of immunosuppressive cells. Notably, the association between immune microenvironment differences and epigenetic regulation suggests that epigenetic alterations may influence disease progression by modulating the immune microenvironment, opening new avenues for therapeutic strategies that target epigenetic regulation to enhance anti-tumor immunity (Wang et al., 2024; Zhou et al., 2024; Ding et al., 2024).

This study has validated previous epigenetic research on PDAC in multiple aspects while also presenting some innovative findings. Consistent with previous studies, our research reaffirms the importance of epigenetic regulation in PDAC development, aligning with earlier research results. For instance, we observed generally high levels of histone modifications and DNA methylation in PDAC cells, which is consistent with Lomberk et al.'s findings that KRAS mutations can promote PDAC progression by regulating histone and DNA modifications (Lomberk et al., 2019). The innovative aspects of our study are mainly reflected in the following areas: First, we employed single-cell RNA sequencing technology, enabling us to study PDAC epigenetic characteristics at the single-cell level, revealing similarities and differences in epigenetic regulation across different cell types. This high-resolution analysis method provides a new perspective for understanding PDAC cellular heterogeneity. Second, by integrating various bioinformatics methods, including hdWGCNA and WGCNA, we systematically identified key epigenetic regulatory genes and pathways. This comprehensive analysis approach allows us to understand more fully the role of epigenetic regulatory networks in PDAC. Lastly, we innovatively linked epigenetic features with the immune microenvironment and drug sensitivity, providing new insights for PDAC immunotherapy and personalized medication.

Compared to existing PDAC prognostic models, our epigenetic feature-based prognostic model has several significant advantages. First, most previous PDAC prognostic models are primarily based on gene mutations or transcriptome data (Chen et al., 2021; Zhang et al., 2015), while our model focuses on epigenetic features, providing a new dimension for prognosis assessment. The importance of epigenetic regulation in tumor progression is increasingly recognized, so our model may capture some important information overlooked by traditional models. Second, our model demonstrates good predictive performance across multiple independent datasets, with AUC values exceeding 0.7 for 1-year, 3-year, and 5-year prognosis predictions. The stability and generalizability of our model highlight the potential of epigenetic features in PDAC prognosis prediction. Additionally, our model not only provides prognostic predictions but also combines them with immune microenvironment and drug sensitivity analyses, giving it greater potential in guiding individualized treatment decisions (Lomberk et al., 2016). In contrast, many existing models primarily focus on prognosis prediction, lacking direct applications for treatment guidance.

Although this study provides many valuable findings, some limitations remain. First, our analysis is primarily based on bioinformatics methods, requiring further experimental validation and mechanistic exploration. Second, while our prognostic model performs well, it still needs validation in larger independent cohorts. Most importantly, the drug sensitivity tests include many drugs not yet clinically applied, necessitating pharmacological research and clinical verification. Future research should focus on in-depth study of the causal relationship between epigenetic regulation and PDAC progression, integrate multi-omics data to construct a more comprehensive PDAC molecular typing system, and investigate the dynamic relationships between epigenetic regulation and PDAC immune microenvironment and drug responses. Based on this, large-scale generalization studies and drug application exploration integrating multi-center data should be conducted.

In conclusion, this study has made significant contributions to epigenetic research in PDAC through multi-level, multi-faceted analysis. We revealed cellular heterogeneity and epigenetic characteristics of the PDAC microenvironment at the single-cell level, systematically identified key epigenetic regulatory genes and pathways, and constructed a high-performance prognostic prediction model based on epigenetic features. Additionally, we uncovered close connections between epigenetic regulation and PDAC mutation characteristics, immune microenvironment, and drug sensitivity. These findings not only deepen our understanding of PDAC molecular mechanisms but also provide new insights for precision medicine, individualized treatment, and immunotherapy optimization. Our research opens new directions for PDAC diagnosis, prognosis assessment, and treatment strategies, potentially advancing clinical practice and ultimately improving patient prognosis and quality of life. However, translating these findings into clinical applications requires further validation and large-scale prospective studies. Overall, this study lays an important foundation for epigenetic research and precision medicine development in PDAC, demonstrating the enormous potential of epigenetics in cancer research.

## Data Availability

The original contributions presented in the study are included in the article/supplementary material, further inquiries can be directed to the corresponding author.
