# Corrigendum: The role of epigenetic regulation in pancreatic ductal adenocarcinoma progression and drug response: an integrative genomic and pharmacological prognostic prediction model

**DOI:** 10.3389/fphar.2025.1560529

**Published:** 2025-02-18

**Authors:** Kang Fu, Junzhe Su, Yiming Zhou, Xiaotong Chen, Xiao Hu

**Affiliations:** Department of Hepatobiliary Pancreatic Surgery, The Affiliated Hospital of Qingdao University, Qingdao, China

**Keywords:** pancreatic ductal adenocarcinoma, epigenetic regulation, single-cell RNA sequencing, machine learning, prognostic model, tumor microenvironment, drug sensitivity

In the published article, there was an error in Figure 13 as published. Due to a data format inconsistency between CSV and TXT files during analysis, an undetected empty row in the header resulted in a misalignment of data. This affected the gene names on the y-axis and cell types on the x-axis, causing a one-row shift in visualization, which led to incorrect matching of color representation with corresponding samples. The corrected Figure 13 and its caption appear below.

**FIGURE 13:**
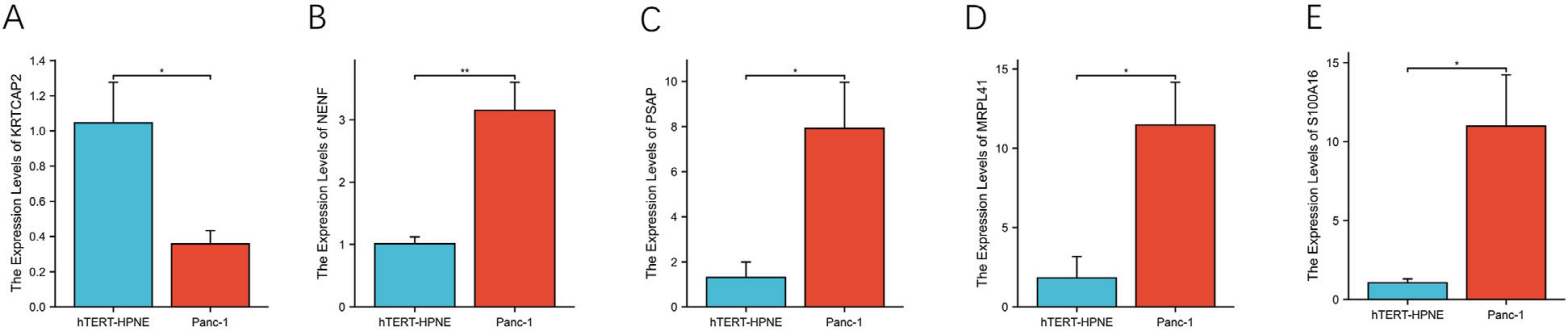
Differential expression analysis of prognostic genes in pancreatic cell lines **(A–E)**. The relative mRNA expression levels of KRTCAP2, NENF, PSAP, MRPL41, and S100A16 were quantified by qRT-PCR in pancreatic cancer cell line PANC-1 and normal pancreatic epithelial cell line hTERT-HPNE.

The authors apologize for this error and state that this does not change the scientific conclusions of the article in any way. The original article has been updated.

